# Antioxidants help favorably regulate the kinetics of lipid peroxidation, polyunsaturated fatty acids degradation and acidic cannabinoids decarboxylation in hempseed oil

**DOI:** 10.1038/s41598-020-67267-0

**Published:** 2020-06-29

**Authors:** Anubhav Pratap Singh, Farahnaz Fathordoobady, Yigong Guo, Anika Singh, David D. Kitts

**Affiliations:** Food, Nutrition, and Health, Faculty of Land & Food Systems. The University of British Columbia, 2205 East Mall., Vancouver, BC V6T 1Z4 Canada

**Keywords:** Biochemistry, Biological techniques, Biophysics, Biotechnology

## Abstract

The seed of the hemp plant (*Cannabis sativa* L.) has been revered as a nutritional resource in Old World Cultures. This has been confirmed by contemporary science wherein hempseed oil (HSO) was found to exhibit a desirable ratio of omega-6 and omega-3 polyunsaturated fatty acids (PUFAs) considered optimal for human nutrition. HSO also contains gamma-linoleic acid (GLA) and non-psychoactive cannabinoids, which further contribute to its’ potential bioactive properties. Herein, we present the kinetics of the thermal stability of these nutraceutical compounds in HSO, in the presence of various antioxidants (e.g. butylated hydroxytoluene, alpha-tocopherol, and ascorbyl palmitate). We focussed on oxidative changes in fatty acid profile and acidic cannabinoid stability when HSO was heated at different temperatures (25 °C to 85 °C) for upto 24 h. The fatty acid composition was evaluated using both GC/MS and ^1^H-NMR, and the cannabinoids profile of HSO was obtained using both HPLC-UV and HPLC/MS methods. The predicted half-life (DT50) for omega-6 and omega-3 PUFAs in HSO at 25 °C was about 3 and 5 days, respectively; while that at 85 °C was about 7 and 5 hours respectively, with respective activation energies (E_a_) being 54.78 ± 2.36 and 45.02 ± 2.87 kJ/mol. Analysis of the conjugated diene hydroperoxides (CDH) and *p*-Anisidine value (*p*-AV) revealed that the addition of antioxidants significantly (*p* < 0.05) limited lipid peroxidation of HSO in samples incubated at 25–85 °C for 24 h. Antioxidants reduced the degradation constant (*k*) of PUFAs in HSO by upto 79%. This corresponded to a significant (*p* < 0.05) increase in color stability and pigment retention (chlorophyll *a*, chlorophyll *b* and c*a*rotenoids) of heated HSO. Regarding the decarboxylation kinetics of cannabidiolic acid (CBDA) in HSO, at both 70 °C and 85 °C, CBDA decarboxylation led to predominantly cannabidiol (CBD) production. The half-life of CBDA decarboxylation (originally 4 days) could be increased to about 17 days using tocopherol as an antioxidant. We propose that determining acidic cannabinoids decarboxylation kinetics is a useful marker to measure the shelf-life of HSO. The results from the study will be useful for researchers looking into the thermal treatment of hempseed oil as a functional food product, and those interested in the decarboxylation kinetics of the acidic cannabinoids.

## Introduction

The nutritional value of hemp seed, a by-product of the plant fibre industry, is attributed to both a high-quality protein content (25%), containing all essential amino acids, and a high-quality lipid content (>30%) which includes all essential fatty acids, and also gamma linoleic acid (GLA) (FAO/WHO). Hempseed oil (HSO) is generally obtained by cold pressing of the seed, which contains a rich source of essential polyunsaturated fatty acids (PUFA). The ratio (2.1:1 to 3:1) of linoleic acid (18:2, ω-6) to alpha-linolenic acid (18:3, ω-3) in HSO provides a balanced fatty acid substrate for downstream n-6 and n-3 eicosanoid production^1^, respectively; critical reactions known for maintaining cell membrane structure and regulating prostaglandins and leukotrienes synthesis pathways. These bioactive agents are responsible for ensuring a balance between homeostasis and physiological mechanisms that include anti-/pro-aggregation, vasodilation, and anti/pro-inflammatory properties^2–4^. In this regard, Canada has recently paid attention to the production of legal industrial hemp that contains less than 0.3% of Δ‐9‐tetrahydrocannabinol (ΔTHC) as well as hemp seed oil (HSO)^5,6^.

Thermal degradation and oxidation are two reactions that produce undesirable changes in edible oils that carry over through processing and storage, and which can result in changes in safety, sensory, and the nutritive value of the oil. Hence, information regarding oxidative and thermal stability of the HSO is indispensable for maintaining quality control for applications that involve its use as an ingredient in food formulations^7^, or as an ingredient in numerous cosmetic, nutraceutical and functional food products^8,9^. Various methods, including determination of primary oxidation (e.g. peroxide value, conjugated dienes) and secondary oxidation (e.g. *p*-Anisidine value, TBARS, and headspace volatile) are commonly used to monitor the oxidation stability of edible oils and also to predict the shelf-life^10^. As lipid oxidation is a relatively slow process at room temperatures, accelerated storage study performed using higher temperatures (i.e. 60–90 °C) is often performed^11^. Arrhenius plots are used to predict oxidative stability and shelf-life of a food product^12^. Also of particular interest are the cannabinoid compounds, with typically higher concentrations of non-psychoactive cannabinoids, including cannabidiolic acid (CBDA) and cannabidiol (CBD), compared to psychoactive compounds tetrahydrocannabinolic acid (THCA), and tetrahydrocannabinol (THC). Although these compounds are present in only small quantities, they have medical interest due to their bioactive anti-convulsive, anti-epileptic, and anti-microbial effects^8^. Acidic cannabinoid acids such as CBDA convert to corresponding neutral forms through a decarboxylation reaction that is catalyzed by heat. Hence, the changes of CBDA/CBD ratio in HSO can be considered as a useful indicator for monitoring HSO storage life.

Numerous studies^13,14^ have evaluated the potential of using different antioxidants to delay auto-oxidation reactions, in edible oils, particularly when exposed to high temperature processing. However, the specific effect of antioxidants on thermal-induced degradation kinetics of omega-6 and omega-3 fatty PUFAs, along with simultaneous changes of cannabinoids has not been reported.

In a previous work^15^, we demonstrated the efficacy of natural plant extracts (rosemary, sage and thyme) in inhibiting the formation of hydroperoxides and preservation of vitamin E levels and omega-3 fatty acid profile during high temperature processing and storage of hempseed and soybean oils. The current study aims to establish the kinetic parameters for modelling of fatty acids degradation and acidic cannabinoids decarboxylation of hempseed oil. We also evaluate the pigment content and correlate it with color changes, study the effect of heat treatment on lipid peroxidation levels, evaluate the fatty acid concentration and identify the cannabinoids present in the HSO. Further, we assess how these kinetic parameters are regulated in the presence of 3 different antioxidants (butylated hydroxytoluene, alpha-tocopherol, and ascorbyl palmitate) in an attempt to reduce the degradation of both PUFAs (ω-6 and ω-3 fatty acids) and acidic cannabinoids in HSO.

## Materials and methods

### Materials

Unrefined cold-press hempseed oil (HSO) was purchased from Manitoba Harvest Hemp Foods (Winnipeg, Manitoba, Canada). All solvents and chemicals used were analytical and/or HPLC grade.

### Oxidative stability and quality of thermally-induced hempseed oil

HSO samples were prepared by adding three individual antioxidants including BHT (positive control) in USDA legislated level of 0.01% w/w, alpha-tocopherol (α-T), and ascorbyl palmitate (AP) at concentrations of 0.02% w/w and 0.02% w/w respectively. Considering the molecular weight of α-T (430.72 g/mol) and AP (414.53 g/mol), which is about twofold that of BHT (220.4 g/mol), when added in the similar weight to BHT, they provide approximately half reactive moles. Hence, to provide the same molar ratio, the amount of 0.02% w/w were chosen for both natural antioxidants (α-T and AP). The samples incubated at 40, 55, 70 and 85 °C for 24 h together with original HSO (negative control). The samples were retrieved at regular time intervals for determination of pigment content (Chlorophyll *a*, Chlorophyll *b* and carotenoids), and color properties (ΔE*) along with conjugated diene hydroperoxides (CDH), *p*-Anisidine value (*p*-AV), fatty acids profile and cannabinoids analysis (CBDA). All experiments were performed in triplicate. Results were compared with samples stored at 25 °C for 15 days (360 h).

#### Chlorophyll a, Chlorophyll b and carotenoids contents

Chlorophyll *a* and *b*, and total carotene were determined according to Aladić *et al*.^16^. HSO samples were dissolved in diethyle ether (pure solvent) in the ratio of 1/50 (w/v) in an ultrasonic bath following by homogenizing for 30 seconds and centrifuging at 3000 rpm for 10 minutes. Using an UV-Vis spectrophotometer (Varian Cary, 50 MPR Microplate Reader, USA), absorbance of the supernatant was measured at 400–700 nm. Chlorophyll *a* represented the maximum absorbance at 660 nm, chlorophyll *b* at 642.5 nm, and the total carotene at 470 nm. All tests were performed triplicate. The concentration of pigments (μg/g) was calculated on the basis of Lambert-Beer Law using Eqs. (1–3).

1 $$Chlorophyll\,a=10.5\,{{\rm{A}}}_{660}-0.97\,{{\rm{A}}}_{642.5}$$

2 $$Chlorophyll\,b=16.36\,{{\rm{A}}}_{642.5}-2.43\,{{\rm{A}}}_{660}$$

3 $$Total\,carotene=(1000\,{{\rm{A}}}_{470}-0.52\,Ch{l}_{a}-36.75\,Ch{l}_{b})/205$$

The amount of each pigment in the HSO (μg/g) was calculated using Eq. (4):

4 $${\rm{C}}={\rm{C1}}\cdot {\rm{V}}\cdot {\rm{D}}/{\rm{G}}$$

where: C = amount of pigment in HSO ((μg/g); C1 = concentration of pigment (mg/L); V = initial volume (mL); D = dilution (if any); G = oil mass (g).

#### Color changes (ΔE*)

The color properties of HSO samples were assessed by by LabScan XE spectrophotometer (HunterLab, VA, USA) equipped with EasyMatch QC software based on the International Commission on Illumination (CIE L*a*b*) method. Lightness (L*), redness (a*) and yellowness (b*) attributes were directly measured by system. Then, the total color change of the samples (ΔE*) was defined by the total distance between two points in three-dimension CIE L*a*b* of color space.

#### Conjugated diene hydroperoxides (CDH) test

Conjugated diene hydroperoxides (CDH) associated with primary products of oxidation were measured according to AOCS standard method 2.501 (AOCS 1998). An aliquot of hemp seed oil sample was dissolved in 5 mL cyclo-hexane and the absorbance of solution was measured at 234 nm using spectrophotometer (Varian Cary, 50 MPR Microplate Reader, USA). Results (g hydroperoxides per 100 g oil) were reported based on the linoleic acid molar absorptivity as Eq. (5):

5 $$CDH(g/100\,g\,oil)=1.0769\times A/C\,(g\,oil/100\,ml\,solution)$$

where: CDH = the value of Conjugated diene hydroperoxides; A = the absorbance of the sample at 234 nm; C = concentration

#### *p*-Anisidine Value (p-AV)

The *p*-Anisidine value (*p*-AV) for determination of secondary oxidation products was measured according to AOCS Official Method Cd 18–90 (2017). HSO samples (2.0 g) were dissolved in 25 ml *n*-hexane and the absorbance of this solution was measured at 350 nm against *n*-hexane using spectrophotometer (Varian Cary, 50 MPR Microplate Reader, USA). Then, one ml of 0.25% *p*-Anisidine in acetic acid (w/v) was added to 5 ml of the solution and kept in the dark for 10 min. The absorbance of the sample solution was measured at 350 nm against the control test containing 1 ml of *p*-Anisidine solution and 5 ml *n*-hexane. All tests were performed in triplicate.

The *p*-AV was calculated according to Eq. (6)^15^:

6 $$p-AV=25(1.2Ab{s}_{1}-Ab{s}_{2})/m$$

where: *p*-AV = the value of *p*-Anisidine; Abs_1_ = the absorbance of the sample solution after 10 min reaction in the dark; Abs_0_ = the initial absorbance of the sample solution; *m* = the amount of HSO (g) used in the analysis.

#### Analysis of fatty acids profile

*Sample Preparation*: The fatty acid profile was determined by producing methyl esters with potassium hydroxide 2M in methanol and using gas chromatography (GC-FID) system^17^. An amount of 3 ml heptane was added to 0.10 ± 0.0 g HSO in a 15 ml test tube and shaken using vortex for 20 s. Then, the sample was saponified with adding 500 μL of methanolic potassium hydroxide (KOH) solution (2M) and vortexed for another 20 s. HCL (2N) was used for eliminating the excessive amount of KOH. The sample was left until the upper layer became clarified. The supernatant comprising of fatty acid methyl esters (FAMEs) was decanted and passed through 0.45 μm filters before injection.

*Gas-FID Chromatography (GC) condition*: (FAMEs) were analyzed by a GC-17A Shimadzu (Shimadzu, Scientific Instruments, Inc., Columbia MD) equipped with a flame ionization detector (FID), Omegawax™ 320 (30 m × 0.32 mm ID × 0.25 µm film thickness) fused silica capillary column and Shimadzu Class-VP Software. The initial column temperature was set at 165 °C for 10 min followed by increasing to the final temperature of 200 °C with a rate of 1.5 °C/min. The injector and detector temperature were 210, and 250 °C, respectively. The FAMEs were detected based on the comparison of their retention time to that of the matched peaks of a mixture of fatty acid methyl ester (FAMEs) standard.

*GC/MS condition*: A Perkin Elmer system of GC-MS (model: Clarus 680-GC - SQ8T Mass Spec.) equipped with TurboMass Ver. 2.3 (NIST 2011) software was used for detection of HSO fatty acids with some changes. Helium (99.99%) at a constant flow rate of 1 ml/min was used as carrier gas. Initial temperature was set at 150 °C holding for 2 min following by increasing to 185 °C with 1.5 °C/min and reached to 220 °C with a rate of 5.0 °C/min A volume of 1 μl prepared sample was injected at 250 °C in a split mode (50:1). MS conditions included ionization energy: 70 eV, ion source temperature: 250 °C, and the mass-to-charge (*m/z*) range: 20–450 atomic mass units. Identification of the FAMEs was performed by comparison of their mass spectra and retention times with corresponding data from FAMEs standard.

#### H^1^ NMR analysis

For NMR studies, the sample was prepared by dissolving 200 μL of hemp seed oil in 800 μL of CDCl_3_. ^1^H spectra for CDCl_3_ solutions were recorded at 600 MHz, on a BRUKER AVANCE 600 (with CRYOPROBE). All the data was analyzed by MestReNova.

#### HPLC-UV and LC-MS analysis

In order to identify the cannabinoids existing in hempseed oil samples, LC-MS analyses were carried out according to Citti *et al*.^18^ using an Agilent 1290 Infinity/6530 Accurate Mass Q-TOF equipped with MassHunter Workstation software B.07.00. The column was Agilent Zorbax Eclipse Plus C18, 2.1 × 50 mm, with 1.8 μm pore size. Mass spectrometer was operated in dual ionization mode (ESI+ and ESI−). The dry gas at a flow rate of 12 ml/min and the nebulizer (N_2_) with pressure of 60 psi at 400 °C and skimmer voltage of 65V were used. The capillary voltage was set at 4.0 kV. The injection volume was 2.00 μL and the mass spectrometer was performed in the range of 50–1000 *m*/*z*. Employing the *m*/*z* corresponding to the molecular ions [M + H]^+^, extracted ion chromatograms (EICs) were acquired.

After identifying the cannabinoids composition of hempseed oil samples by LC-MS system, HPLC analyses of cannabinoids were conducted on an Agilent Technologies system series 1100 (Agilent, USA) composed of a quaternary pump, an autosampler, a column heater, and a diod array detector (DAD). Cannabinoids were separated by C18 column (Zorbax, 3.5 μm, 4.6 mm × 150 mm, Agilent, USA) and acquired at 288 nm. The mobile phase was composed of A) water and B) acetonitrile both with 0.1% formic acid as the buffer in a gradient of 10–100% B for the first 10 min followed by isocratic elution with 100% B for 5 min and re-equilibration at 20 min to the first condition. The mobile phase was pumped at a flow rate of 1.0 ml/min. The column temperature was set at 20 °C. Three injection in volume of 5 μL were performed for each sample.

### Kinetic studies

#### Degradation of fatty acids

The degradation kinetics of essential fatty acids (ω-3 linoleic acid and ω-6 α-linolenic acid) were assessed by incubating HSO samples with/without antioxidants at different levels of temperature (40 to 85 °C) for 24 h. The same procedure was used for samples stored at 25 °C for two weeks. The degradation of ω-6 and ω-3 fatty acids followed single first-order kinetic as Eq. (7):

7 $${C}_{t}={C}_{0}{{\exp }}^{-kt}$$

Where, C_t_ = concentration at time *t*, *C*_0_ = initial concentration, *e* = base e, *k* = rate constant of degradation (1/h), *t* = time.

The rate constant (*k*) was calculated based on the slope of the *Ln* of fatty acids (ω-6, ω-3) retention (%) vs. time (*h*) plot. Then, the time needed for 50% and 90% degradation (DT50 or half time and DT90) were determined by Eqs. (8) and (9):

8 $$DT50=\frac{Ln2}{k}$$

9 $$DT90=\frac{Ln10}{k}$$

By using Arrhenius plot between the logarithmic values of *k* (*Lnk*) versus 1/*T*(*K*^−1^), the value of the slope corresponds to −*Ea*/*R* where *E*_*a*_ is the activation energy and *R* is the universal gas constant equal to 8.31441 J*mol^−1^*K^−1^.

#### Decarboxylation of CBDA

The decarboxylation kinetics of CBDA and its conversion to CBD were studied by heating HSO samples containing antioxidants as well as control sample at 70 and 85 °C for 24 h. The decarboxylation of CBDA which followed single first-order kinetics was assessed using Eq. (7). The DT50 and DT90 of the samples were also evaluated using Eqs. (8) and (9).

### Statistical analysis

The experimental data are specified as mean ± SD of three tests. Data were analyzed statistically using Minitab software ver. 18.0 (Pennsylvania, USA). A one-way analysis of variance (ANOVA) followed by Tukey’s test was applied for statistical analysis of data at *p* < 0.05.

## Results and discussion

### Color changes (ΔE*) of thermally-induced hemp seed oil with and without antioxidants and its relation to pigment content

The total color change, expressed as ΔE* of HSO samples during heating at temperatures between 40 to 85 °C and at different intervals during 24 h period is presented in Supplemental Table 1. Results of the control samples, incubated at 25 °C, in dark glass bottles, for 15 days are presented in Supplemental Fig. 1.

Color stability of the HSO improved when antioxidants were added (see Supplemental Table 1). Obón *et al*.^19^ had shown that color differences that ranged from 0 to 1.5 do not denote significant visual changes; however, when ranges are greater than 5, significant differences can be distinguished. Using this parameter, we observed a marked change in ΔE* (6.41) and a yellowish tint in heated HSO samples, relative to controls, when incubated at 85 °C, starting at 8 h and continuing to 24 h. These thermally induced color changes in HSO are likely due to generation of aldehydes, that have known maximum absorption at 350 nm. The color changes of HSO that contained BHT, α-T or AP did not exceed the distinguishing value (>5) when incubated at 40 °C and 55 °C, respectively. HSO that was stored at 25 °C for 15 days expressed changes in ΔE* that were same as samples supplemented with antioxidants (Supplemental Fig. 1).

**Figure 1:**
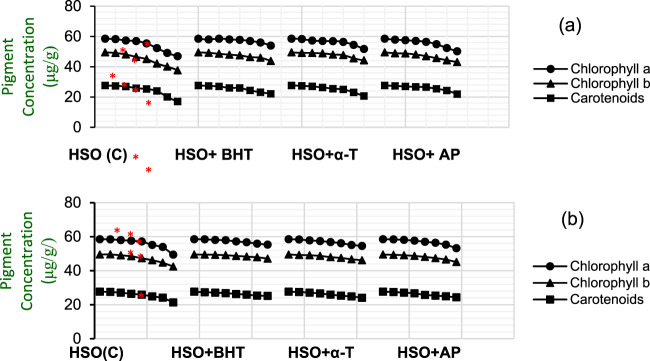
Changes in pigment contents (Chlorophyll a, Chlorophyll b and Carotenoids) of HSO samples during 24 h heat treatment: 85 °C (**a**) 70 °C (**b**). *Significant difference to samples with antioxidant (p < 0.05).

The color change of HSO samples could be related to the conversion of chlorophyll *a* to brownish pheophytins, which are known to occur when exposed to heat^20^. Heat treating of HSO at 70 °C and 85 °C produced a significant (*p* < 0.05) loss in total carotenoid content during storage (Fig. 1). While, chlorophyll *a* is responsible for blue-green color of vegetables, chlorophyll *b* gives a yellow-green shade in oils. Analysis of Chlorophyll *a* and *b* in this study confirmed that chlorophyll *a* degraded more rapidly than chlorophyll *b* as the temperature increased (Fig. 1). Losses of carotenoids in these samples suggest that reactions with the carotenoid polyene chain and primary lipid oxidation products occurred to produce radicals yielding carotenoids-adducts^21^. Similar results have been obtained^22,23^ with beta-carotene during heating of red palm olein and olive oil, respectively; over a range (e.g. 50–100 °C). The results for carotenoids reduction obtained in the current study is also in agreement with Kadian *et al*.^21^, who reported a 61–64% decrease in β-carotene carrot extract when heated at 70 °C and 80 °C, respectively.

### Effect of heat treatment on HSO peroxidation reactions

**Figure 2:**
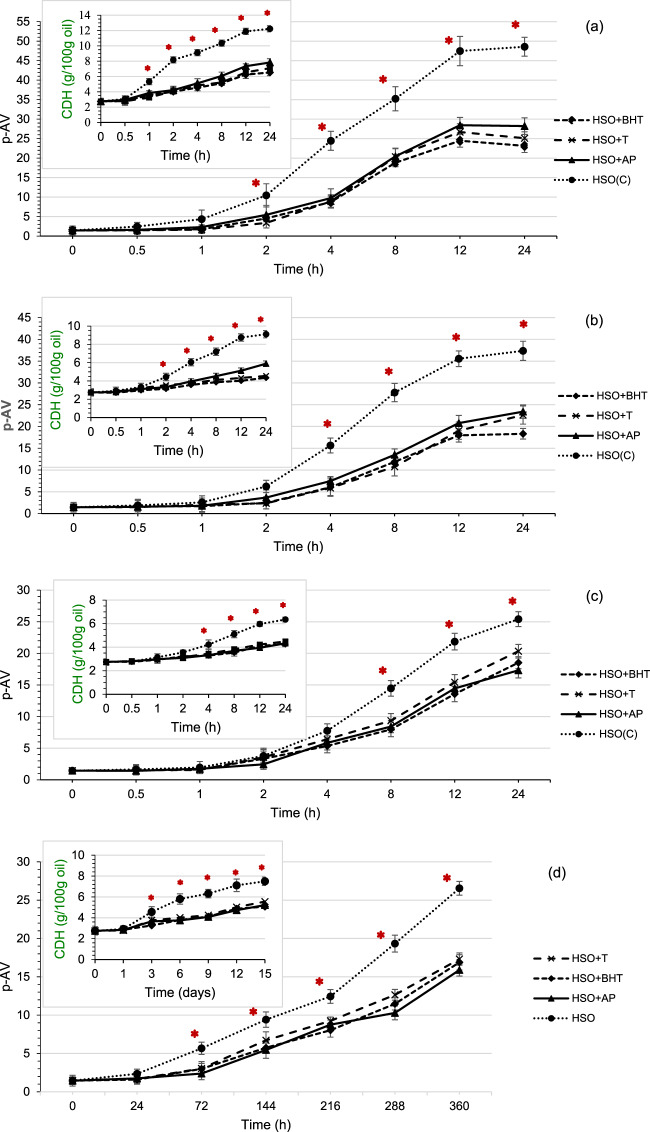
Oxidative status (p-AV and CDH) of hempseed oil samples incubated at different temperatures: 85 °C (**a**), 70 °C (**b**), 55 °C (**c**), and 25 °C (**d**) *Significant difference to antioxidant containing samples (*p* < 0.05).

In general, HSO is considered to be an unstable oil due to the high proportion of α-linoleic acid and γ-linolenic acid, both of which are susceptible to oxidization during heating and storage^24^. The results of CDH and *p*-AV, depicting the peroxidation state of HSO samples both with and without antioxidants including BHT (positive control), α- tocopherol (α-T) and ascorbyl palmitate (AP) in samples incubated for 24 h at temperatures ranging from 40 to 85 °C are shown in Fig. 2. This figure also includes the CDH and p-AV values of HSO samples that were stored at room temperature (25 °C) for 2 weeks; for a comparison of the accelerated shelf-life study performed under ambient temperature conditions. Conjugated dienes (CDH) measurement tracks thermal oxidation reactions during the early (primary) stages of oxidation; while Anisidine value (*p*-AV) is useful for evaluating the secondary products of lipid oxidation. As linoleic acid is the predominant fatty acid of HSO, *p*-AV gives an indication of aldehyde production rate in HSO^25^. CDH and *p*-AV values in control HSO samples were 2.75 and 1.45, respectively, but these values increased significantly (*p* < 0.05) after 24 h heat treatment. The increasing rate for *p*-AV reached to a plateau after 12 h heating at 85 °C. No increase or plateau in *p*-AV was reached when samples were stored at room temperature for 2 weeks. A significant increase (*p* < 0.05) in *p*-AV value of control sample was observed when heated beyond 8 h, 4 h and 2 h at 55 °C, 70 and 85 °C, respectively.

Adding antioxidants to HSO produced a reduction in *p*-AV by 39–53% in samples incubated at temperature ranges from 55–85 °C. The relative inhibitory effect of antioxidants on *p*-AV were in the order of BHT > α-T > AP at 70 °C and 85 °C, respectively and BHT > AP > α-T at 55 °C. Bag and Chattopadhyay^26^ also observed that the inhibition in lipid peroxidation attributed to BHT was 23.2% higher than α-T when added in similar amount (200 µg/ml). We obtained a relatively better inhibitory effect of α-T, compared to *p*-AV at elevated heating temperatures, which translates to a potential for prolonged storage. The results of CDH and p-AV for the samples stored at 25 °C also revealed that after 15 days (360 h), oxidation occurred in HSO as evidenced by the p-AV value exceeding a score of 20. Adding antioxidants stabilized HSO samples to acceptable levels (reduced p-AV from 27 to 15–17) (Fig. 2), producing the following relative order of inhibition: AP > BHT > α-T. Our finding is similar to other researchers, wherein the addition of AP at 400 ppm in both cotton seed and virgin olive oils, respectively during storage at 60 °C for 28 days resulted in significant reduction of PV^27^. At elevated temperatures, the addition of antioxidants maintained p-AV values within 20 for 55 °C and 70 °C, and to within 30 for 85 °C. In general, α-T was relatively more effective at stabilizing HSO compared to ascorbyl palmitate, albeit both were comparable to BHT. α-T was also found to be an efficient antioxidant when used at concentrations that ranged from 50–200 ppm for delaying the oxidization of sunflower triacylglycerols at 55 °C^28^. α-T is also relatively more effective than ascorbyl palmitate in delaying oxidation in salad dressing enriched with fish oil during 6 weeks storage at room temperature^29^. It is important to note that BHT, which was the positive control used in this study, consistently provided a better inhibition of *p*-AV values as compared to α-T. The mechanisms underlying BHT and α-T activity to retard lipid oxidation is by hydrogen donation to lipid peroxyl radicals, which leads to interference in the chain propagation, or initiation stage of lipid oxidation. Antioxidant capacity of AP occurs as a result of scavenging oxygen and thus changing the redox property to a reduced state, that regenerates primary antioxidants, such as tocopherol isomers^27^,^30^.

### Fatty acids evaluation of HSO

#### GC-FID/GC-MS analysis

The fatty acid profiles and content in HSO determined by GC-FID and GC-MS analysis are presented in Table 1. In this study, the most abundant fatty acid in HSO was linoleic acid (60.52 ± 0.63%) followed by α-linolenic (18.44 ± 0.12%), oleic (6.63 ± 0.11%) and palmitic acid (6.06 ± 0.09%). Previous studies^11^,^18^,^31^,^32^ reported higher amounts of oleic acid (9.56–11.98%) and lower concentration of linoleic acid (55.05–59.77%). In our study, we also detected a small but significant amount of stearidonic acid (0.93 ± 0.01%), which compared to the amount reported by Cardillo^33^ in HSO and hulled hemp seed samples. In this study, the degree of unsaturation (91.12%) and the ratio of ω-6/ω-3 fatty acids (3.3/1) remained in agreement with the values reported by other researchers^11^,^32^.

**Table 1:** Fatty acid composition of hempseed oil (*Cannabis sativa* L.) detected and analyzed by GC-MS and GC-FID.

Fatty acid	*m/z[M + H]^+^	Retention time (min)	**Mean ± SD (%)
Palmitic acid (C16:0)	256.4	4.53	6.06 ± 0.09
Stearic acid (C18:0)	248.48	6.25	2.41 ± 0.03
Oleic acid (C18:1, ω_9_)	282.47	6.65	6.63 ± 0.11
Linoleic acid (C18:2, ω_6_)	280.44	7.50	60.52 ± 0.63
γ -linolenic acid (C18:3, ω_6_)	278.43	7.87	4.33 ± 0.08
Alpha-linolenic acid (C18:3, ω_3_)	278.43	8.46	18.44 ± 0.12
Stearodonic acid (C18:4, ω_3_)	276.4	8.98	0.93 ± 0.01
Eicosenoic acid (C20:1, ω_9_)	310.51	9.23	0.38 ± 0.02
Decosanoic acid (C22:0)	340.58	9.85	0.30 ± 0.01
PUFA			84.11
MUFA			7.01
Saturated fatty acids/Unsaturated fatty acids			0.09
ω_6_/ω_3_			3.3/1

*GC-MS results are based on employing the *m*/*z* corresponding to the molecular ions [M + H]^+^ **Fatty acid values are the means ± SD (n = 3) of GC-FID peak area percentage (%).

#### ^1^H-NMR analysis

We also used NMR to confirm both the PUFA and saturated fatty acid content of HSO. The fatty acid compositions and the ratios among them were determined from the areas from the characteristic signals of each fatty acyl chain (See Supplemental Fig. 2). We confirmed former findings^18^, that at the main composition of fatty acids in HSO include linolenic acid (ω-3), linoleic acid (ω-6), oleic acid (ω-9) and saturated fatty acid. The ratio between linoleic acid (ω-6): linolenic acid (ω-3) and linolenic acid (ω-3): oleic acid (ω-9) was measured to be 3.12 and 2.16, respectively. These data are consistent with the findings obtained using the GC method indicating that NMR analysis enabled ratio configuration of different fatty acids.

### Cannabinoid profile of HSO

LC-MS/HPLC-UV and GC/GC-MS have been typically used for identification and quantification of cannabinoids in hempseed oil^34–36^. Using LC-MS and comparing with a mixture of cannabinoids standards in this study, seven cannabinoid compounds including Cannabidivarin (CBDV), CBDA, CBD, Cannabigerol (CBG), and small amounts of Tetrahydrocannabivarin (THCV), THCA and THC (<0.2%) were detected (Table 2). Pellegrini *et al*.^36^ reported that HSO’s THC concentration (25.0 ± 0.5 ng/ml) was considerably higher than CBD (3.7 ± 4.2 ng/ml) and CBN (2.4 ± 0.3 ng/ml), respectively. Citti *et al*.^34^ found more CBDA, CBD and CBN than THCA and THC. In contrast to these other works^34,36^, CBN was not detected in our HSO sample.

**Table 2:** Retention times, peak area (%) (HPLC-UV) and MS spectrometric data (LC-MS) of cannabinoids detected in hempseed oil.

Cannabinoid compound	*m/z[M + H]^+^	Retention time (min)	**Cannabinoid composition (%)
CBDV	287.19	6.35	2.13 ± 0.04
CBDA	359.22	6.81	61.61 ± 2.98
CBG	317.24	6.95	15.77 ± 1.54
THCV	286.41	7.01	0.45 ± 0.05
CBD	315.23	7.12	19.73 ± 1.04
THC	315.23	7.85	0.13 ± 0.01
THCA	359.22	8.15	0.19 ± 0.02

*LC-MS results are based on employing the *m*/*z* corresponding to the molecular ions [M + H]^+^ **Cannabinoid composition values are the means of peak area percentage (%) of HPLC chromatograms ± SD (n = 3).

### Kinetic studies

#### Degradation of omega-6 and omega-3 fatty acids

**Figure 3:**
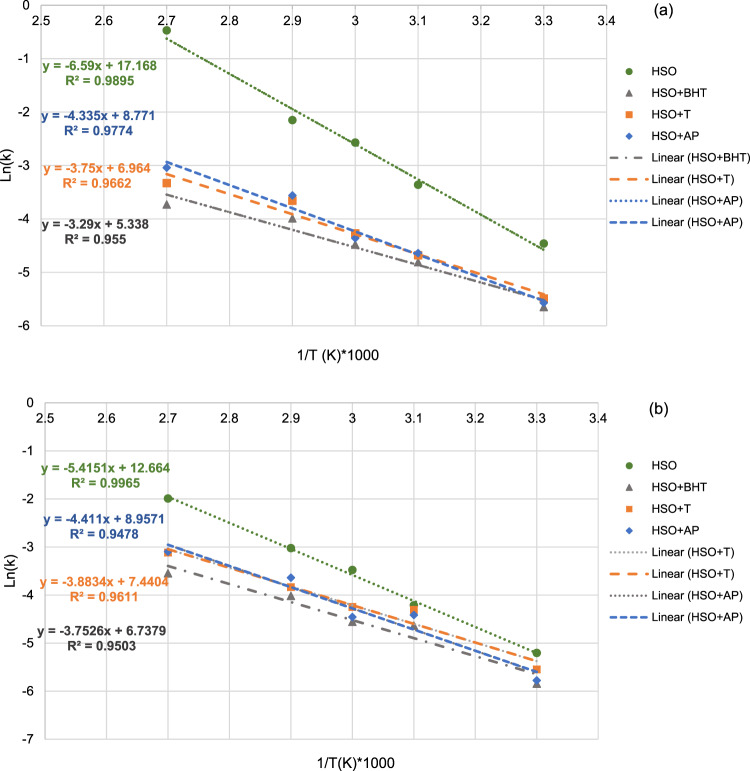
Arrhenius plots for calculation of Ea and the extrapolation of (k) at temperatures of 25, 40, 55, 70, and 85˚C: omega-6 fatty acids (**a**) and omega-3 fatty acids (**b**).

The results for degradation rate constant (*k*), DT50 and DT90 of total omega-6 and total omega-3 fatty acids present in HSO incubated at 25–85 °C for 24 h are shown in Table 3. The Arrhenius plots based on natural logarithm of the degradation constant rate (Ln *k*) of omega-6 and omega-3 fatty acids in HSO, versus the inverse of the temperature (1/T °K) displayed a first order linear relationship (*R*^2^ value of 0.9478 to 0.9895) (Fig. 3). A rise of 90.07–95.81% in the rate constant (*k*) occurred on increasing the temperature from 25 to 85 °C in HSO, without addition of antioxidant, showing that degradation of omega-6 and omega-3 fatty acids were dependent on temperature. However, adding different antioxidants led to a drop in rate constant (*k*) to 78.93%. At 25 °C, the predicted half-life (DT50) and DT90 of HSO, without antioxidant were 74.21 h and 256.51 h for omega-6 fatty acids and 125.45 and 418.18 h for omega-3 fatty acids, respectively; thus indicating higher stability of omega-3 fatty acids at this temperature. Increasing the temperature to 85 °C resulted in a greater resistance to omega-6 fatty acids degradation compared to that of omega-3 fatty acids; according to higher DT50 and DT90 values. At this temperature, the samples with antioxidants added showed improved DT50 and DT90 for both omega-3 and omega-6 fatty acids. In this regard, BHT acted more efficiently followed by α-T and AP.

**Table 3:** Degradation rate constant (*k*), half-life (DT50), degradation time for 90% loss (DT90) and activation energy (*E*_*a*_) of total omega-6 and total omega-3 fatty acids of HSO incubated at 25–85 °C for 24 h.

Incubation temperature (°C)	Sample	K (h^−1^)		DT50 (h)		DT90 (h)		*R*^2^	
		ω-6 ω-3		ω-6 ω-3		ω-6 ω-3		ω-6 ω-3	
25	*HSO*	0.0093	0.0055	74.21	125.45	256.51	418.18	0.94	0.87
	*HSO* + *BHT*	0.0035	0.0029	197.54	237.93	656.24	793.1	0.93	0.94
	*HSO* + *T*	0.0041	0.0039	166.23	176.92	552.22	589.74	0.96	0.95
	*HSO* + *AP*	0.0038	0.0031	182.17	222.58	605.17	741.93	0.93	0.93
40	*HSO*	0.0157	0.0148	44.13	46.62	146.60	155.40	0.92	0.93
	*HSO* + *BHT*	0.0081	0.0097	79.21	71.13	263.13	237.11	0.94	0.96
	*HSO* + *T*	0.0092	0.0135	70.37	51.11	233.77	170.37	0.93	0.92
	*HSO* + *AP*	0.0096	0.0121	72.09	57.02	239.49	190.08	0.94	0.91
55	*HSO*	0.0210	0.0309	32.86	22.33	109.15	74.43	0.94	0.96
	*HSO* + *BHT*	0.0113	0.0105	60.98	65.71	202.56	219.04	0.93	0.92
	*HSO* + *T*	0.0139	0.0143	49.52	48.25	164.50	160.83	0.95	0.98
	*HSO* + *AP*	0.0127	0.0116	54.53	59.48	181.14	198.27	0.95	0.92
70	*HSO*	0.0728	0.0487	9.47	14.16	31.59	47.22	0.98	0.90
	*HSO* + *BHT*	0.0184	0.0180	37.15	38.33	123.41	127.77	0.95	0.93
	*HSO* + *T*	0.0256	0.0216	26.15	31.94	86.87	106.48	0.95	0.90
	*HSO* + *AP*	0.0283	0.0263	24.47	26.23	81.29	87.45	0.96	0.92
85	*HSO*	0.1013	0.1367	6.81	5.04	22.70	16.82	0.96	0.98
	*HSO* + *BHT*	0.0293	0.0288	23.10	23.95	76.75	79.86	0.94	0.98
	*HSO* + *T*	0.0356	0.0444	19.80	15.54	65.78	51.80	0.98	0.96
	*HSO* + *AP*	0.0478	0.0453	14.43	15.23	48.11	50.77	0.96	0.98
**E**_**a**_ **(kJ/mol)**									
**HSO**		**HSO + BHT**			**HSO + T**			**HSO + AP**	
ω-6	ω-3	ω-6	ω-3		ω-6		ω-3	ω-6	ω-3
54.78 ± 2.36	45.02 ± 2.87	27.35 ± 1.18	31.17 ± 2.45		30.41 ± 2.09		32.28 ± 1.97	35.97 ± 2.56	36.66 ± 2.43

Data for K(h^−1^), DT (50), and R^2^ are the average of three replications Data for E_a_ (kJ/mol) values are the means ± SD (n = 3).

Activation energy represents the sensitivity of the compound to degrade due to thermal treatment. In this study, higher *E*_*a*_ indicated more susceptibility of the omega-6 and omega-3 fatty acids to the thermic changes. All tested HSO samples were less stable at higher temperature (70 and 85 °C) compared to the lower temperatures (25,40 and 55 °C). Original heat-treated HSO had an *E*_*a*_ value of 54.78 and 44.97 (kJ/mol) for omega-6 and omega-3 fatty acids, respectively, signifying the lower resistance to heat from omega-3 fatty acids (Table 3). These values are 1.22–2.02 times more than when HSO had antioxidants added, thus showing the effect of antioxidants to reduce thermal oxidation reactions in HSO. This finding was lower than *E*_*a*_ for oxidation of HSO previously found at 70, 80 and 90 °C (69.97 kJ/mol) by other researchers^11^. They^11^ also reported an exponential relationship between the rate of lipid oxidation and temperature. In our current study, adding antioxidants was more effective in decreasing the activation energy of omega-6 fatty acids than omega-3 s. Incidentally, AP was less effective compared to BHT and α-T. This can be related to the heat liability of ascorbic acid and its esteric compounds with a first-order thermal degradation kinetics^37^.

#### Decarboxylation of cannabidiolic acid (CBDA)

**Figure 4:**
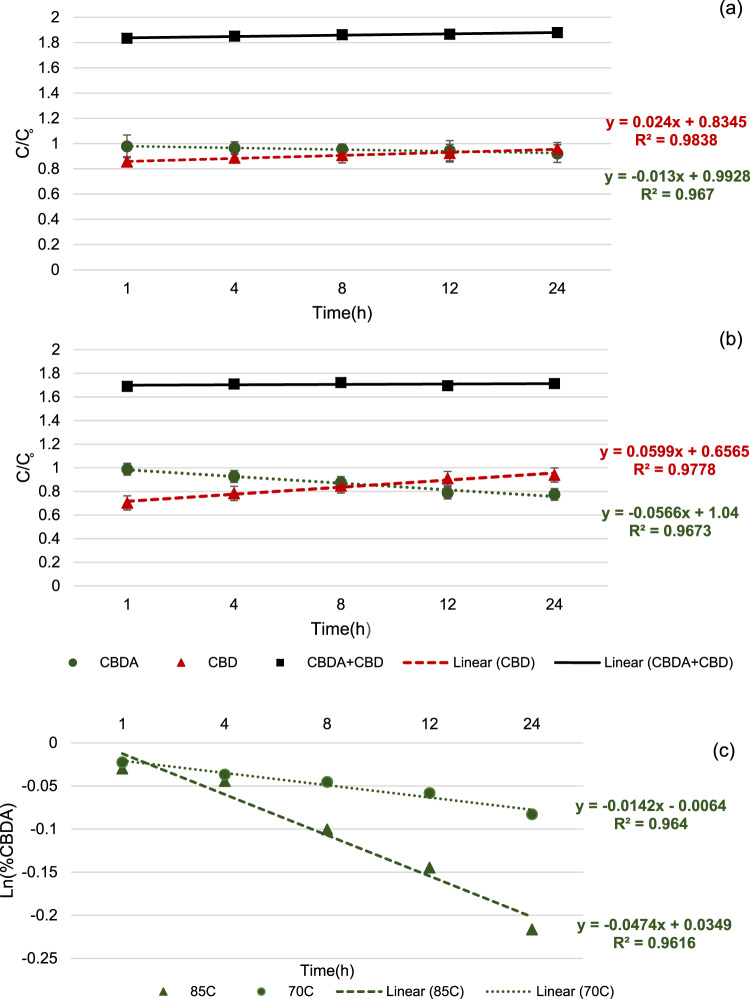
Decarboxylation of CBDA: 70 °C (**a**) and 85 °C (**b**); Kinetics for decarboxylation of CBDA at 70 and 85 °C (C).

In order to study the influence of heat treatment (e.g. 70 and 80 °C for 24 h) on the stability of acidic cannabinoids in HSO, the decarboxylation of acidic cannabinoids was studied. To avoid the evaporation of cannabinoids, decarboxylation studies were conducted in a closed vessel. Due to trace amount of THCA in our HSO sample, CBDA, the most abundant of acidic cannabionod identified in HSO was selected as the chemical marker for decarboxylation studies. The linear regressions of the decarboxylation kinetics of CBDA at temperatures of 70 and 85°C in closed condition as well as the samples with antioxidants incubated at 85°C for 24 h are presented in Fig. 4. The decrease of CBDA during 24 hours incubation of HSO samples at 70 and 85 °C, occurred as a result of decarboxylation reaction and consequent increasing in CBD presumed a first order reaction (Fig. 4). At these temperatures, the sum of loss of CBDA and formation of CBD remained constant (the recorded loss of CBDA at 70 and 85 °C was <0.02%). Hence, it was assumed that at these temperature, the decarboxylation of CBDA led only to the production of CBD. From the equations, the kinetic parameters have been calculated and recorded for CBDA loss (Table 4). Comparing our results with those of Citti *et al*.^34^ using CBDA retention in both open and closed condition at temperatures from 80 to 120 °C, the rate constant (*k*) (3.37 ± 0.20 × 10^−5^ and 1.01 ± 0.04 × 10^−4^ at 80 and 90 °C, respectively) was lower than our results. In our study, HSO samples with antioxidants exhibited lower decarboxylation rate of CBDA (Table 4). The presence of hydroxyl groups belonging to antioxidants might decrease the lipid-solubility of CBDA with carboxyl group, leading to protect it from degradation. To have a better understanding of these mechanism, further detailed studies is needed to be performed.

**Table 4:** Decarboxylation rate constant (*k*), half-life (DT50), time for 90% loss (DT90) of CBDA present in HSO samples incubated at 85 °C for 24 h.

Sample	K(h^−1^)	DT50 (h)	DT90 (h)
HSO (control)	0.00777 ± 0.0053^a^	88.80 ± 1.67^a^	298.58 ± 3.76^a^
HSO + BHT	0.00457 ± 0.0044^b^	150.98 ± 1.98^b^	507.65 ± 7.85^b^
HSO + T	0.00169 ± 0.0011^d^	408.28 ± 5.98^d^	1372.78 ± 11.34^d^
HSO + AP	0.00253 ± 0.0012^c^	272.72 ± 3.13^c^	916.99 ± 9.76^c^

Values are the mean es on the same column do not share the same lowercase letters are significantly different at *p* < 0.05.

## Conclusion

Evaluation of oxidative predisposition of HSO exposed to heat treatments with and without the presence of antioxidants (α-Tocopherol, BHT and ascorbyl palmitate) was performed by measuring changes in conjugated diene hydroperoxides (CDH) and p-Anisidine value (p-AV). These chemical markers confirmed the potential of antioxidants to improve the of storage life of HSO. Color changes during heat treatment corresponded to aldehyde formation during lipid peroxidation and/or chlorophyll a degradation. GC-FID/GC-MS followed by ^1^H-NMR analysis of HSO confirmed the ω-6/ω-3 ratio of 3.12 and 3.3 which is suited for human nutrition. Measuring the kinetic parameter (rate constant *k*) for degradation of ω-6 and ω-3 fatty acids, provided the prediction of half-life (DT50) and 90% loss(DT90) of these fatty acids during storage of HSO at 25 °C. Using HPLC-UV and HPLC/MS analysis, cannabidiolic acid (CBDA) was found to be the major cannabinoid compound in HSO. The decarboxylation kinetics of CBDA to CBD provided an estimation for cannabinoid stability. Further studies are needed to have better understanding of correlations between decarboxylation of acidic cannabinoids and stability of PUFAs in the presence of other antioxidants of mixtures thereof, in stabilized HSO.

## Supplementary information

Supplementary Information.
