# Supplementary material for: Antioxidants help favorably regulate the kinetics of lipid peroxidation, polyunsaturated fatty acids degradation and acidic cannabinoids decarboxylation in hempseed oil
Source: Sci Rep. 2020 Jun 29;10:10567. doi: 10.1038/s41598-020-67267-0 (PMC7324387; doi:10.1038/s41598-020-67267-0)
Supplement: Supplementary file 1 — Supplementary Information. [file 41598_2020_67267_MOESM1_ESM.docx]

**Supplemental documents**

**Supplemental Table 1:** Color changes (*ΔE**) calculated for HSO samples during 24 h storage at 40,55,70 and 85°C

| **Sample** | Temperature (°C) | **Color difference value (*ΔE**)** | | | | |
| --- | --- | --- | --- | --- | --- | --- |
|  |  | **2h** | **4h** | **8h** | **12h** | **24h** |
| **HSO** | 40 | 1.29^a^ | 1.50^a^ | 2.23^ab^ | 3.69^c^ | 3.98^c^ |
|  | 55 | 1.35^a^ | 2.15^ab^ | 4.08^c^ | 4.15^c^ | 6.11^d^ |
|  | 70 | 2.09^a^ | 2.87^ab^ | 6.25^c^ | 6.99^cd^ | 8.54^e^ |
|  | 85 | 3.12^a^ | 4.67^b^ | 6.41^c^ | 7.80^d^ | 10.41^e^ |
| **HSO+BHT** | 40 | 0.63^a^ | 1.78^bc^ | 2.86^c^ | 3.96^cd^ | 4.95^d^ |
|  | 55 | 1.19^a^ | 1.57^a^ | 2.97^ab^ | 4.02^bc^ | 5.13^c^ |
|  | 70 | 1.28^a^ | 1.94^ab^ | 3.56^b^ | 5.08^c^ | 5.52^c^ |
|  | 85 | 1.24^a^ | 2.45^ab^ | 4.12^bc^ | 5.28^c^ | 5.86^c^ |
| **HSO+T** | 40 | 0.78^a^ | 1.50^a^ | 2.34^ab^ | 2.89^b^ | 4.42^c^ |
|  | 55 | 1.66^a^ | 2.95^ab^ | 3.86^b^ | 4.39^bc^ | 5.49^c^ |
|  | 70 | 2.76^a^ | 3.95^ab^ | 4.69^bc^ | 5.54^c^ | 6.21^d^ |
|  | 85 | 1.33^a^ | 2.79^ab^ | 4.58^bc^ | 5.73^c^ | 6.48^d^ |
| **HSO+AP** | 40 | 0.83^a^ | 1.76^a^ | 2.98^ab^ | 4.70^bc^ | 4.95^c^ |
|  | 55 | 1.07^a^ | 1.88^ab^ | 3.06^bc^ | 4.53^c^ | 5.09^c^ |
|  | 70 | 1.93^a^ | 3.55^b^ | 4.64^bc^ | 5.32^c^ | 5.54^c^ |
|  | 85 | 2.15^a^ | 3.09^ab^ | 5.01^c^ | 5.47^c^ | 6.31^d^ |

Values represents means (n=3)

^a-e^ In a row followed by the different small letters are significantly different by Turkey’s test (*p* < 0.05)

cd

bc

a

c

b

e

d

cd

g

f

f

i

i

ij

g

j

k

jk

h

l

lm

m

lm

d

Supplemental **Fig.** **1** Color changes (*ΔE**) of HSO samples (without/with antioxidant) during 15 days storage

at 25°C

^a-m^ Bars depicited with different small letters are significantly different by Turkey’s test (*p* < 0.05)


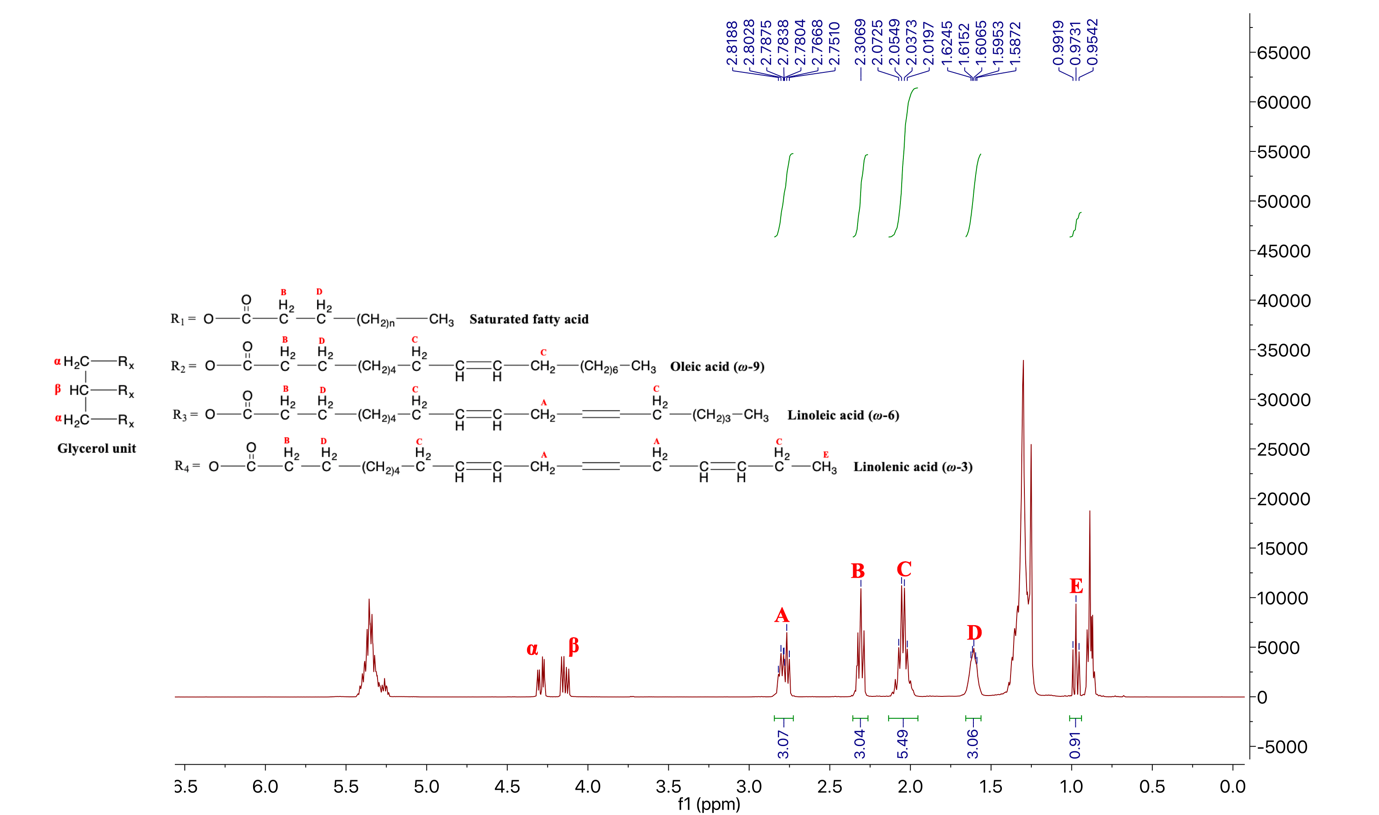


Supplemental **Fig. 2** ^1^H NMR spectrum of [hemp seed](https://www.sciencedirect.com/topics/biochemistry-genetics-and-molecular-biology/hemp-seed) oil (HSO) in CDCl_3_

**Supplemental Fig. 3** Decarboxylation of CBDA in HSO samples (without/with antioxidants) incubated at 85°C for 24h

List vof abbriviations:

ANOVA: Analysis of Variances

AOAC: Association of Official Analytical Chemists

AP: Ascorbyl palmitate

BHT: Butylated hydroxytoluene

CBD: Canabidiol

CBDA: Cannabidiolic acid

CBDV: Cannabidivarin

CBG: Cannabigerol

CDH: Conjugated diene hydroperoxides

CIE: International Commission on Illumination

E_a_: Activation energies

ESI: Electrospray Ionization

GC-FID: Gas Chromatography with Flame Ionization Detector

GLA: Gamma linoleic acid

HPLC: High Performance Liquid Chromatography

HSO: Hempseed oil

LC-MS: Liquid chromatography–Mass Spectrometry

NMR: Nuclear Magnetic Resonance

*p*-AV: *para-*Anisidine value

PUFAs: Poly unsaturated fatty acids

TBARS: Thiobarbituric acid reactive substance

THC: tetrahydrocannabinol

THCA: tetrahydrocannabinolic acid

THCV: Tetrahydrocannabivarin

UV-Vis : Ultra Violet Visible

α-T: Alpha-tocopherol
